# After COVID-19 vaccinations: what does living and working in nursing homes look like?

**DOI:** 10.1186/s12877-023-03987-y

**Published:** 2023-05-16

**Authors:** Judith H. J. Urlings, Ramona Backhaus, Hilde Verbeek, Bram de Boer, Raymond T.C.M. Koopmans, Debby L. Gerritsen, Jan P.H. Hamers

**Affiliations:** 1 Department of Health Services Research, Care and Public Health Research Institute, Maastricht University, Postbus 616, 6200 MD Maastricht, the Netherlands; 2 Living Lab in Ageing and Long-Term Care, Maastricht, the Netherlands; 3 Department of Primary and Community Care, Radboud University Medical Center, Nijmegen, the Netherlands; 4 De Waalboog “Joachim en Anna”, Center for Specialized Geriatric Care, Nijmegen, the Netherlands

**Keywords:** COVID-19, Nursing homes, Vaccination, Policy, Visitor restrictions

## Abstract

**Background:**

Nursing homes were disproportionally affected by the COVID-19 pandemic. Vaccination was considered critical for the normalization of daily live of nursing home residents. The present study investigates the impact of the prolonged COVID-19 pandemic and the effect of vaccinations on the daily lives of residents and staff in Dutch nursing homes.

**Setting and participants:**

The sample consisted of 78 nursing homes that participated in the Dutch national pilot on nursing home visits after the COVID-19 pandemic. One contact person per nursing home was approached for participation in this mixed-methods cross-sectional study.

**Methods:**

Data was collected twice through questionnaires in April and December 2021. Quantitative questions focused on recent COVID-19 outbreaks, progress of vaccination, effects of vaccination on daily living in the nursing home and burden experienced by staff. Open-ended questions addressed the prolonged effect of the pandemic on residents, family members and staff.

**Results:**

The overall vaccination rate of residents across nursing homes appeared to be high among both residents and staff. However, daily living in the nursing home had not returned to normal concerning personal interactions, visits, the use of facilities and work pressure. Nursing homes continued to report a negative impact of the pandemic on residents, family members and staff.

**Conclusions:**

Restrictions to the daily lives of residents in nursing homes were stricter than restrictions imposed on society as a whole. Returning to a normal daily living and working was found to be complex for nursing homes. With the emergence of new variants of the virus, policies strongly focusing on risk aversion were predominantly present in nursing homes.

## Background

Across the world, nursing homes, who provide care for the most vulnerable people within a society, were disproportionally affected by the COVID-19 pandemic [1]. An international study based on 22 countries worldwide estimated that 41% of COVID-19 deaths were nursing home residents [1]. In the Netherlands, approximately 50% of all COVID-19 deaths in the first wave of the pandemic occurred in nursing homes [2].

Visiting bans and restrictive measures to prevent and control the spread of COVID-19 have negatively affected the well-being of residents, their families and staff [3, 4]. International research shows that visitor bans negatively impacted the mood and behavior of residents severely leading to an increased use of psychotropic medication and increased feelings of guilt, fear, worry and isolation in residents’ families [5].

The mission of nursing home care to support residents to autonomously shape their daily lives [6] stands in sharp contrast to restrictions and social isolation measures [7]. Finding a balance between infection prevention and residents’ autonomy is crucial for meeting the nursing homes’ mission to support residents in leading their lives as independently as possible. Considerations for this balance have changed, as more experience with the coronavirus was gained and COVID-19 vaccinations became widely available.

The availability of COVID-19 vaccinations is considered to be critical for minimizing the impact of the pandemic [8]. Nursing home residents and their professional caregivers were among the first to be vaccinated in the Netherlands and many other countries [9]. Initial studies from the period immediately following the first vaccinations indicate a drastic drop in infections and deaths due to COVID-19 in nursing homes after vaccination [10–12]. High vaccination rates, widely available personal protective equipment and testing capacity [13, 14] should allow nursing homes to respond to COVID-19 outbreaks differently than in earlier waves of the pandemic. As vaccinations and early detection will protect residents from spread of COVID-19 and severe disease, one may expect that fewer restrictions would be imposed on nursing home residents and that daily living would return to normal.

The present study therefore investigates the long-term impact of COVID-19 vaccinations on residents, family members and staff in Dutch nursing homes. The study focusses on vaccination of residents and staff, and the impact of these vaccinations on the daily living and work in nursing homes.

## Methods

### Setting and sample

This study is part of a long-term monitoring study focusing on the impact of the COVID-19 pandemic and restrictions to prevent the spread of COVID-19 on nursing home residents, their family members and nursing home staff [15–17]. Nursing homes in the Netherlands provide long-term care to frail (mostly elderly) people with complex care needs. Somatic (for residents with physical disabilities) and psychogeriatric wards (for residents with, most often, dementia) can be distinguished in nursing homes.

The sample consists of 78 nursing homes that participated in the Dutch national pilot [15, 17]. Of those, 26 nursing homes re-opened for visitors on May 11 2020, 50 nursing homes were allowed to welcome back visitors as of May 25 2020. These 76 nursing homes were representative for the Netherlands, as they were randomly selected from all local health authority regions in the Netherlands. Two nursing homes were additionally included in this monitor from April 2021 on their own request. Descriptive data (number of beds, number of currect residents) are avaible in Table 1 of our previously published study [17]. In each nursing home, one contact person was appointed to fill out electronic questionnaires. The nursing homes selected a person (staff member) they considered able to provide the most information about the specific nursing home. Often, contact persons were nursing home managers or local quality or policy officers.

### Data collection

Data was collected through two electronic questionnaires, the first one in April 2021 and the second in December (Qualtrics Research Suite XM), using personalized links. Questionnaire 1 (April) included the following topics:

- infections in the nursing home;
- progress of the vaccination campaign of residents and staff;
- effects of vaccination on the daily lives of nursing home residents and staff;
- compliance with protective measures;
- plans to further relax protective measures within the nursing home;
- effect of the prolonged COVID-19 pandemic and protective restrictions on residents, family members and staff;

Questionnaire 2 (December) included the following topics:

- infections in the nursing home;;
- progress of the booster vaccination campaign of residents and staff;
- compliance with protective measures;
- effect of the prolonged COVID-19 pandemic and protective restrictions on residents, family members and staff;
- sick leave & experienced work pressure by staff.
- pressure and support experienced by nursing homes.

Both questionnaires consisted of mainly closed ended questions (yes/no), with open text fields for respondents to provide additional illustrations and information. The impact of the prolonged COVID-19 pandemic on residents, family and staff was assessed by open-ended questions.

- What is the impact of the long-standing COVID-19 pandemic on residents/loved ones of residents/staff of this nursing home?

Questionnaire 1 was sent on March 23 2021. After two reminders, we closed the survey on April 7. Questionnaire 2 was sent on December 3 2021. After three reminders, we closed the survey on December 16.

### Data analyses

Descriptive statistics were calculated for quantitative data. Data on open-ended questions were analyzed thematically and discussed by the research team.

### Ethical considerations

The study was conducted in accordance with Dutch law and the Declaration of Helsinki. The study protocol (2020–6549) was reviewed by the local Medical Ethics Review Committee “CMO Regio Arnhem Nijmegen,” which concluded that the study was not subject to the Medical Research Involving Human Subjects Act. Information about the study was provided per e-mail to the respondents. Participation was voluntarily. Respondents gave informed consent to participate in the study and could withdraw from participation at any moment.

## Results

Of the invited 78 respondents, 59 (76%) filled in the questionnaire in April and 57 (73%) in December.

### Progress in vaccination rates

#### Residents

Vaccinations were available for nursing homes residents and staff in the Netherlands from January 2021. Across all nursing homes, respondents estimated that 77% of residents were fully vaccinated in April. In 71% (n = 42) of the nursing homes, the estimated percentage of fully vaccinated residents was over 80%. On the other hand, 20% (n = 12) of the nursing homes estimated that less than 60% of residents had been fully vaccinated.

Explanations for a lower vaccination rate included: nursing homes waiting to receive vaccines for the (planned) second dose, fragile health (e.g. receiving palliative care, very short life expectancy), family members did not consent to vaccination, fear of side effects and low confidence in the vaccine.

In December 2021, the overall percentage of fully vaccinated residents (2 vaccinations) was 93%. At that time, 89% of nursing homes (n = 51) had started offering booster vaccinations for residents. On average, 77% of residents had received their booster vaccination.

#### Staff

63% (n = 37) of respondents indicated that they had a reliable impression of the vaccination rate of the staff of their nursing home in April. Nursing homes estimated that on average 65% of their nursing home staff were vaccinated. However, the vaccination rate varied greatly between nursing homes. In 19% of the participating nursing homes (n = 11), the contact person estimated that more than 80% of staff had been vaccinated. In 20% (n = 12) of nursing homes, the vaccination rate was estimated to be below 60%. The most important reasons for staff not to get the vaccine included: pregnancy/wish to become pregnant/breastfeeding, fear of side effects and fear of long-term consequences related to vaccination.

In December 2021, nursing homes estimated that 80% of staff was fully vaccinated (two vaccinations). 77% of nursing homes (n = 44) had started offering booster vaccinations for staff. On average, 39% of staff had received their booster vaccination.

### Infections in the nursing homes

In April, 11 nursing homes (19%) reported current infections among residents or staff in their nursing home. In December, 35 nursing homes (61%) reported current infections. In case of an outbreak, 17% of nursing homes (n = 10) banned visitors from the entire nursing home in April. In December, this was the case in 7% of nursing homes (n = 3). Almost half of the nursing homes banned visitors from the unit where the outbreak took place in April (48%, n = 28), while 38% (n = 16) did so in December. Non-infected residents could decide whether they wanted to receive visitors in 58% (n = 34) and 81% (n = 34) of nursing homes in April and December respectively.

The 4th wave is significantly different from the previous waves. The mortality rate is much lower and residents have far fewer complaints than before the vaccinations.

Respondent quote. December 2021.

### Impact of vaccinations on the daily lives of nursing home residents

After the initial vaccination campaign in April 2021, most nursing homes allowed residents to have contact with other residents (85%, n = 50). In most nursing homes, residents were able to take part in group activities with other residents (76%, n = 45). In 51% of nursing homes (n = 30) the level of assistance provided by volunteers had come back to the pre-pandemic level.

The majority (71%, n = 42) of nursing homes indicated that changes in their visiting policy were implemented after the vaccination campaign in April 2021. Although visitors were allowed in all nursing homes, the nature of visits had not normalized. In the majority of nursing homes, visitors were not allowed to hug their relatives (90%, n = 53), could not enter the general living areas (73%, n = 43), were not allowed to be in contact with visitors of other residents (85%, n = 50), and could not stay over for dinner (85%, n = 50) or sleep over (80%, n = 47). The nursing home restaurant was open to visitors in a minority of nursing homes (19%, n = 11).

Additional detailed information on policies in specific day-to-day situations related to visiting, and resident interactions in April 2021 is presented in Tables 1 and 2.

**Table 1:** Policies regarding interactions between residents & visitors (April & December)

	APRIL		DECEMBER	
Activity	Yes (#nursing homes)	No	Yes (#nursing homes)	No
Most residents have contacts with other residents	50	9		
Family members/visitors can hug the resident	6	53	29	28
Visitors are not required to wear a facemask	3	56		
Visitors can stay in shared living rooms	12	47		
Visitors have contacts with visitors of other residents	16	43		
Visitors can stay for dinner	9	50		
Visitors can sleep over	12	47		
Visitors can bring a pet	39	20		
Visitor no longer need to register for a visit	8	51		
Visitors are supervised by nursing home staff	8	51		

**Table 2:** Policies regarding visitors after vaccination campaign (April & December)

	APRIL		DECEMBER	
Activity	Yes (#nursing homes)	No	Yes (#nursing homes)	No
For most residents, visits proceed as before the pandemic	7	52		
Most residents receive more than one visitor	46	13		
Most residents receive more than one visitor per visit	30	29		
Most residents receive their grandchildren as visitors	34	25	51	6
There is no limit to the number of visitors a resident can receive	3	56	8	51
Residents can receive visitors any time of the day	43	16	48	9
COVID-19 free residents can leave the nursing home to make visits to family and friends			51	6

This situation persisted in December 2021: almost half of the nursing homes did not permit physical contact between resident and visitor (49%, n = 28), some nursing homes banned school aged children from visiting (11%, n = 6) or only allowed visitors within visiting hours that were installed since the pandemic (16%, n = 9). In more than half of nursing homes visitors where limited to a maximum of four per day (56%, n = 32). Fifteen nursing homes (29%) allowed only one or two visitors per day. Detailed information on policies related to visiting in December 2021 is presented in Tables 1 and 2.

In 36% of nursing homes (n = 21), the extent to which services in the nursing homes could be used changed after the initial vaccination campaign in April. In most nursing homes, outdoor facilities could be used again (71%, n = 42). In almost all nursing homes, residents received external services such as provided by a hairdresser (100%, n = 59) and received care by care providers such as occupational therapists (97%, n = 57).

Additional detailed information on policies in specific day-to-day situations related to activities and nursing home facilities is presented in Table 3.

**Table 3:** Policies regarding facilities and activities in the nursing home (April)

Activity	Yes (#nursing homes)	No
The nursing home restaurant is open for visitors	11	48
The nursing home restaurant is open for staff	22	37
The nursing home restaurant is open for people from the community	9	50
Outdoor spaces of the nursing home are freely accesible for residents & visitors	42	17
Most residents get the usual care from professionals caregivers (e.g. physiotherapist)	57	2
Most residents receive services from the hairdresser, pedicure etc.	59	0
Most residents are assisted by volunteer workers as before	30	29
Most residents can take part in activities again	53	6
Most residents can take part in group activities with other residents	45	14
Visitors can take part in activities	3	56
The nursing home organized activities in collaboration with external parties	2	57
Most residents can leave the nursing home if they wish to do so (e.g. for a walk)	59	0
Most residents can leave the nursing home to make visits to family and friends	46	13

In April about half of the respondents reported that they had a plan to relax protective measures and return to a more normal daily living in the nursing home (n = 27). Those contact persons mostly referred to nursing home specific protocols or organization wide policy plans. Still, a quarter (n = 15) of the respondents reported that they did not have such plans at that point. The remaining respondents (n = 17) described that they would follow guidelines provided by others/authorities (i.e. National Government, National Institute for Public Health & Environment (RIVM), branch associations for organizations active in long-term care or local outbreak management teams).

### Effect of the prolonged restrictions on residents, family and staff

#### Residents & family members

A majority of respondents reported decreased well-being and increased loneliness in residents due to the COVID-19 pandemic in April 2021. According to respondents, the number of social interactions for residents was lower compared to the period before the pandemic. Social activities could only take place in small groups, lowering the social contact that residents experienced with other residents. Additionally, the number and frequency of visitors from outside the nursing home was lower than before the pandemic. Lastly, the loss of co-residents to COVID-19 had had a big impact on residents.

“Residents of the nursing home also miss the usual buzz in the house….

Our nursing home was forced to become a kind of inward-looking community.”

Respondent quote. April 2021.

Almost unanimously, respondents indicated that the prolonged corona pandemic had a negative effect on family members in April. Family members visited less often, or with less people at the same time. Social distancing measures, the use of facemasks and not being able to celebrate special opportunities together (e.g. religious holidays, birthdays) was hard for family members. Additionally, family members missed social contact with others in the nursing home (e.g. family members of co-residents, staff).

### Staff

According to the majority of respondents, the prolonged pandemic had a negative effect on staff in both April and December. In April, respondents described that work pressure, fatigue and stress increased while relaxation opportunities in staffs’ free time decreased. More colleagues were on sick leave/mandatory quarantine, and staff had to cope with negative reactions of visitors on protective measures, continuously changing protective measures, and the loss of residents.

### Sick leave & workpressure in staff

In December, the average estimated percentage of staff on sick leave in the participating nursing homes was 11%. The majority of respondents reported the work pressure in their nursing home was (extremely) high (84%, n = 48), and that the work pressure had increased compared to the summer 2021 period (67%, n = 38). Respondents indicated difficulties to arrange minimal staffing (56%, n = 32), to provide day-to day care (58%, n = 33), and to guarantee a normal daily living standard for residents (54%, n = 31). Some nursing homes had to rely on the help of family members (26%, n = 15) or volunteers (19%, n = 11) to provide care for residents.

*“All unnecessary forms of consultation and training have been cancelled to guarantee day-to-day care. Incidentally, people (with a nursing background) from the support services assist in care. The pressure is on the employees who are authorized to perform care tasks.”*

*Respondent quote. December 2021.*

### Compliance with protective measures

In April, respondents gave account of high compliance with protective measures such as the mandatory use of facemasks and social distancing by staff (100%, n = 59) and visitors (98%, n = 58). By December, 18% (n = 10) of respondents reported that compliance with protective measures by staff was lower than at the start of the pandemic. Half of the respondents experienced no difference (56%, n = 32), while 26% (n = 15) found that compliance was higher. For visitors, almost half of the respondents indicated that compliance to protective measures was lower than at the start of the pandemic (45%, n = 26). 47% (n = 27) reported no difference and 7% (n = 4) reported that compliance by visitors was higher.

By December, about half of the respondents reported to experience pressure due to COVID-19 related conflicts and discussion among staff (49%, n = 27), between staff and family members (49%, n = 27) and between staff and residents (14%, n = 8).

*“A small group of informal caretakers does not behave correctly and are verbally aggressive, which has a major impact on employees. Just like the constant discussions they are pulled into.”Respondent quote. April 2021.*

## Discussion

The current study is the first to explore the long-term impact of vaccinations against Covid-19 on daily life of Dutch nursing home residents, their family members and staff.

Estimated vaccination rates of residents and staff appeared to be high. When examining the daily lives of residents in nursing homes however, we noted normal daily living for nursing home residents had not been restored. For staff, the work pressure remained higher than before the COVID-19 pandemic.

The general expectations regarding Covid-19 vaccinations were high: a sufficiently high vaccination rate was believed to lead to the normalization of daily living in nursing homes. This study shows that the expectations regarding vaccinations (i.e. far-reaching relaxation of protective measures and return to normal daily living and working) have not been met for nursing homes. Vaccinations have had a positive effect – the spread of infections in nursing homes is decreased in residents and staff [18] and less residents died due to COVID-19 [12] – but the effect on normalization of daily living was lower than expected. Everyday life in nursing homes still appears to be mostly an internal matter as contact with visitors and people from the community is limited. These results are in line with international findings from e.g., Sweden, where nursing home residents described the everyday life in the nursing home as ‘like living in a bubble’ [19]. Given the lifting of restrictions in the Netherlands in our study period (end of lockdown in May 2021, end of social distancing September 2021 [20]), we see that restrictions imposed on nursing home residents were more strict than those imposed on members of society living independently, despite residents being protected from serious illness by high vaccination rates [10–12]. The level of person-centered care provided has not come back to its pre-pandemic level. For example, many residents are still not allowed to welcome visitors any time of the day. Although far-reaching restrictions in daily living stand in contrast to the mission of nursing homes, it appears that policies focusing on risk avoidance are still predominant in nursing homes.

When examining the daily work of staff in nursing homes, we note that the work pressure is high and nursing homes experience difficulties in providing day-to-day care due to staffing issues. While volunteers were previously active in providing well-being activities, they are now asked to provide basic care in several nursing homes. This at the expense of person centered well-being activities. It can be argued that the COVID-19 pandemic will have a long-term effect on the nursing home care sector.

### Limitations

Some methodological limitations must be considered for this study. First, data was collected from one professional contact person per nursing home with good knowledge of the daily life and policy within the nursing home. Direct data collection with residents, family members and staff might have resulted in a more detailed insight in the impact the prolonged pandemic has on them.

Secondly, the vaccinations rates reported in this study are estimations made by the respondent, and must therefore be interpreted with caution. This is especially the case for vaccination rates of nursing home staff, as employers in the Netherlands are not allowed to register staffs’ vaccination status for privacy reasons. A large proportion of respondents indicated, however, that they had a reliable impression of the vaccination rate of the staff of their nursing home.

### Conclusions & implications

Vaccinations rates among nursing home residents in the Netherlands are high. Nevertheless, this study shows that daily living and working in nursing homes had not normalized to the pre-pandemic state in December 2021. Although the impact of the COVID-19 pandemic and measures to prevent the spread of COVID-19 are felt throughout the entire society [21], this study shows that restrictions imposed on nursing home residents by nursing homes are even more strict. The COVID-19 pandemic and preventive measures continue to have an important negative impact on the lives of residents, family & staff.

The mission of nursing home care is to support residents to autonomously shape their daily lives [6]. Although far-reaching restrictions in daily living stand in contrast to that mission, policies focusing on risk avoidance are still present in nursing homes.Returning to a normal daily living and working is found to be complex for nursing homes. With the emergence of new variants of the virus, policies strongly focusing on risk aversion are predominantly present in nursing homes. Additional motives to adhere to strict local policies might include: lack of national policy that can function as a guidance for nursing homes, the high percentage of staff on sick leave and the (extremely) high work pressure in nursing homes. Shortage of staff might reduce the capacity of nursing homes to assist their residents and their family members in making safe visits and activities possible.

Further monitoring is advised to follow up on the normalization of daily living and working in nursing homes.

## Data Availability

The datasets generated and analyzed in the current study are not publicly available to ensure the privacy of participating nursing homes. Data are available from the corresponding author on reasonable request.
